# Von Hippel–Lindau Disease-Associated Endolymphatic Sac Tumours: Seven Cases and Genotype–Phenotype Features

**DOI:** 10.3390/curroncol32110633

**Published:** 2025-11-12

**Authors:** Qin Wang, Junhui Huang, Zhikai Zhao, Yu Su, Nan Wu, Shiming Yang, Weidong Shen, Na Sai, Weiju Han

**Affiliations:** 1 Senior Department of Otorhinolaryngology Head and Neck Surgery, The 6th Medical Center of Chinese PLA General Hospital, Beijing 100853, China; wqzdb5233@163.com (Q.W.);; 2 State Key Laboratory of Hearing and Balance Science, Beijing 100853, China; 3 National Clinical Research Center for Otorhinolaryngologic Diseases, Beijing 100853, China; 4 State Key Laboratory of Hearing Science, Ministry of Education, Beijing 100853, China; 5 Beijing Key Laboratory of Hearing Impairment for Prevention and Treatment, Beijing 100853, China

**Keywords:** endolymphatic sac tumour, von Hippel–Lindau disease, multidisciplinary team, surgical procedures, genotype–phenotype

## Abstract

Von Hippel–Lindau disease-associated endolymphatic sac tumors (VHL-associated ELSTs) present diagnostic challenges due to their rarity and nonspecific symptoms. This study describes clinical, pathological and genotypic features to guide treatment. We retrospectively analyzed seven patients with VHL-associated ELSTs. The mean age of otologic symptom [hearing loss (100%) and facial nerve paralysis (85.71%)] onset was 22.43 ± 8.68 years (range: 10–33). Surgical management included trans-labyrinthine and subtotal temporal bone resection approaches. Among three patients with severe preoperative facial nerve dysfunction, two underwent great auricular nerve grafting improved to House–Brackmann grade IV, while one receiving hypoglossal–facial nerve anastomosis reached grade V. Genetic testing identified pathogenic *VHL* gene missense mutations in three patients. Two female patients demonstrated disease progression during pregnancy. Literature analysis revealed exon-specific patterns: Exon 1 mutations correlated with cerebellar/spinal hemangioblastomas in female patients, while Exon 3 mutations were associated with multisystem tumors. These findings support that VHL-associated ELSTs manifest early with otologic symptoms and demonstrate exon-specific phenotypic patterns. Optimal management requires complete surgical resection, genetic diagnosis, and a multidisciplinary approach to address these complex tumors and achieve favorable outcomes.

## Simple Summary

Von Hippel–Lindau disease-associated endolymphatic sac tumors are challenging to diagnose due to their rarity and nonspecific symptoms. This study of seven patients found that all experienced hearing loss, often with facial nerve paralysis that began at a young age. Surgical removal was the primary treatment, with postoperative facial nerve function improving through procedures like nerve grafting. Genetic testing revealed that mutations in different parts of the Von Hippel–Lindau gene were linked to clinical features. Notably, two female patients experienced disease progression during pregnancy. This study concludes that optimal patient outcomes rely on surgical management, genetic diagnosis, and collaborative multidisciplinary care.

## 1. Introduction

Von Hippel–Lindau (VHL) disease is a rare autosomal dominant hereditary tumour syndrome that is important for cancer genetics because of its multisystemic manifestations [1]. With an estimated prevalence of 1:30,000–1:40,000 live births [2], it demonstrates age-dependent penetrance and phenotypic variability, validating Knudson’s “two-hit” hypothesis [3]. Germline mutations in the VHL tumour suppressor gene (3p25-26) cause this disease. The VHL protein serves as the substrate-recognition subunit of the VBC-CUL2 E3 ubiquitin ligase complex, targeting hypoxia-inducible factors (HIFs) for proteasomal degradation [4]. This regulation is essential for metabolic homeostasis. VHL protein dysfunction disrupts the normal degradation pathway of HIF-α, leading to its stabilization. This stabilized HIF-α activates the transcription of proangiogenic factors and cell cycle regulators, driving the pathogenesis of VHL-associated neoplasms such as renal cell carcinoma (RCC), central nervous system (CNS) haemangioblastomas, and pancreatic neuroendocrine tumours. Notably, endolymphatic sac tumours (ELSTs) develop in ~25% of patients with VHL disease [5], who typically present with sensorineural hearing loss (SNHL, 100%), tinnitus (92.3%), vertigo (61.5%) and facial nerve (FN) paralysis (7.7%) [6,7]. Progressive tumour growth can erode mastoid air cells, extend into the middle ear and cerebellopontine angle (CPA), and cause symptoms such as FN paralysis, dysphonia, dysphagia, choking, and tongue/sternocleidomastoid atrophy (cranial nerve IX–XII involvement). Despite its clinical significance, research on VHL-associated ELSTs remains limited.

The human inner ear undergoes a highly coordinated morphogenetic process during embryogenesis, beginning with the formation of the otic placode and culminating in the differentiation of cochlear, vestibular, and endolymphatic structures. Among these, the endolymphatic sac (ELS) and duct arise from the dorsal aspect of the otic vesicle around the fifth to seventh week of gestation, establishing a delicate interface between the membranous labyrinth and surrounding dura.

**Figure 1:**
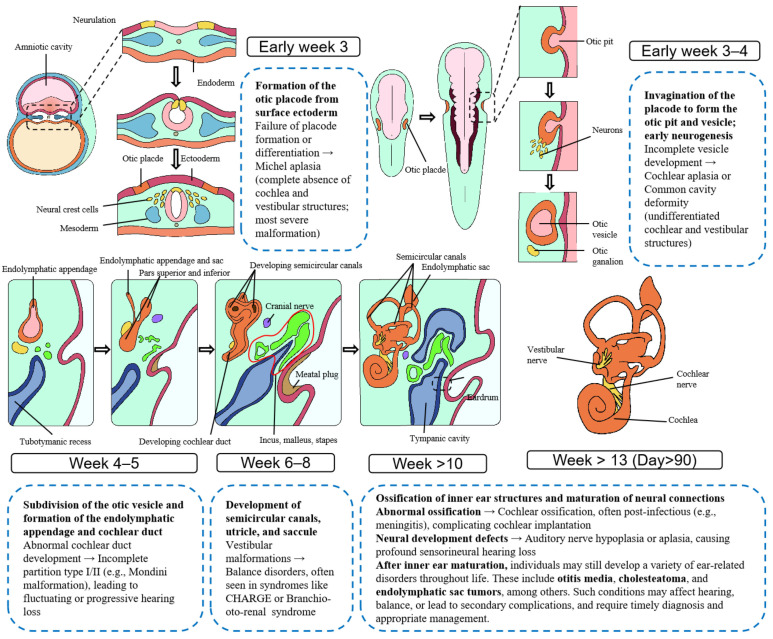
Embryological Development of the Human Inner Ear and Associated Malformations. This figure illustrates the morphological progression of the human inner ear from embryonic week 3 to beyond week 13. It sequentially depicts the formation of the otic placode, invagination and differentiation of the otic vesicle, development of the semicircular canals and cochlea, and establishment of the endolymphatic system. Developmental anomalies and associated disorders are annotated at corresponding stages, providing insight into the temporal and structural origins of congenital inner ear malformations.

Disruptions in this developmental trajectory—whether due to genetic mutations, aberrant epithelial persistence, or local mesenchymal interactions—may predispose to structural anomalies or neoplastic transformation. ELSTs, rare low-grade papillary neoplasms typically arising in the posterior petrous bone, are thought to originate from the epithelium of the ELS. Their anatomical localization and histological features suggest a potential link to embryonic remnants or dysregulated differentiation within the endolymphatic system. Figure 1 presents a comprehensive embryonic schematic of inner ear development.

This report presents our clinical experience with VHL-associated ELSTs, highlighting key diagnostic and therapeutic challenges. By integrating genotype–phenotype features, we demonstrate exon-specific clinical patterns that inform risk stratification, guide genetic counselling, and underscore the importance of individualized, multidisciplinary management strategies for this rare neoplasm.

## 2. Materials and Methods

A retrospective analysis of 7 VHL disease-associated ELSTs patients who underwent ELST resection at our department was conducted. The clinical data included serial clinical examinations, audiometric and radiological evaluations, genetic testing results, and detailed operative records. This study was approved by the Ethics Committee of the Institutional Review Board of the Chinese PLA General Hospital (Ethics Approval No. S2023-468-01). A systematic literature review was performed through PubMed to identify all previously reported genotype-phenotype features in VHL-associated ELST patients.

### 2.1. Audiological, Radiological and Surgical Evaluation

Audiological evaluations were performed during routine assessments or upon symptom changes. Pure-tone audiometry (PTA) was used to measure air and bone conduction thresholds, and hearing was classified on the basis of the 2021 WHO criteria [8]. Perioperative temporal bone high-resolution computed tomography (HRCT) and contrast-enhanced brain Magnetic resonance imaging (MRI) were performed for all patients. The FN function was graded using the House–Brackman (H-B) scale. ELSTs were classified on the basis of intraoperative size and extent using our system [9]: Type-I tumours are small tumours confined to the ELS, possibly with local semicircular canal and/or dural invasion; resection typically involves a transmastoid approach to preserve the integrity of the FN and inner ear. Type-II tumours are large tumours that cause extensive erosion beyond the semicircular canals and dura around the ELS and involve structures such as the tympanum, otic capsule, FN, jugular foramen, internal auditory canal (IAC), CPA, or sigmoid sinus; resection requires subtotal petrosectomy, which is tailored to the tumour extent. Each modality offers distinct advantages in lesion detection, characterization, and surgical planning. Some patients underwent cochlear implantation (CI) with devices incompatible with MRI. Given the genetic nature of the disease and its propensity for recurrence and multisystem tumor development, regular MRI surveillance is essential. Consequently, these patients later required explantation of cochlear implant to facilitate ongoing imaging and disease monitoring. Given the anatomical complexity and variable clinical presentation of ELST in VHL disease, accurate diagnosis often requires a combination of imaging modalities (Table 1).

Intraoperative FN management included decompression, rerouting, great auricular nerve grafting, and hypoglossal-FN anastomosis. Formalin-fixed, paraffin-embedded surgical specimens were sectioned at 4 μm and stained with haematoxylin and eosin (H&E) for routine histopathological evaluation. Immunohistochemical (IHC) staining was performed on selected samples using a tailored panel of antibodies based on clinical and histological context. The antibodies included CK, CK7, CK5, CD31, CD34, D2-40, EMA, GFAP, Ki-67, S-100, Syn, CgA, TTF-1, TG, PAX-8, CD56, Vimentin, and PAS. Not all markers were applied to every case. The Ki-67 proliferation index ranged from 0% to <5%. All slides were independently reviewed by two experienced pathologists. Disease progression was defined as radiological enlargement of lesions and/or worsening of clinical symptoms.

### 2.2. Next-Generation Sequencing

DNA was extracted from peripheral blood samples (2–3 mL). Patients 6 and 7 underwent targeted Sanger sequencing using eight primers designed from the GenBank reference sequence to cover the three-exon open reading frame of the VHL gene. The purified PCR products were subsequently sequenced on an ABI 3130 Avant genetic analyser (Applied Biosystems, Foster City, CA, USA). Patient 1 underwent whole-exome sequencing (WES) via the Illumina platform (MGI Tech Co., Ltd., located in Shenzhen, Guangdong, China), achieving deep coverage (≥500×). All sequencing data were aligned to the GRCh37/hg19 reference genome, annotated using the ClinVar and gnomAD databases, and classified according to ACMG guidelines [10] to ensure precise variant identification and clinical interpretation.

### 2.3. Literature Review and Statistical Analysis

Published ELST cases from 1997 to the present were reviewed by screening the PubMed database using the search terms “endolymphatic sac tumour” AND “VHL mutation”, identifying a total of 75 publications. Studies were excluded if they (1) did not specify VHL mutation positions in ELST patients, (2) lacked histologically confirmed ELST diagnoses, or (3) provided insufficient clinical phenotype details. Following screening, 9 papers met the inclusion criteria [11,12,13,14,15,16,17,18,19]. The study included the following variables: age at diagnosis, sex, family history, VHL mutation type, and the presence of concomitant VHL-related tumours.

## 3. Results

### 3.1. Clinical Data

A total of 75 patients were diagnosed with VHL disease from 2008 to 2025, comprising 41 males and 34 females. The mean age at initial consultation was 38.6 ± 12.5 years (range 13–75). ELSTs were documented in 29 patients, comprising 11 males and 18 females, with a mean initial consultation age of 38.5 ± 15.1 years (range 18–69). A total of 7 patients—2 males and 5 females—presented VHL-associated ELSTs. The mean age at the onset of otologic symptoms was 22.43 ± 8.68 years (range 10–33), with 5 patients demonstrating a familial history. The pathological findings revealed a characteristic papillary-glandular architecture. Three patients received genetic testing, all of whom presented VHL gene mutations: Exon 3 (c.485G>T, c.499C>T) and Exon 1 (c.194C>G).

The clinical manifestations included progressive SNHL (7/7, 100%), FN paralysis (6/7, 85.71%), tinnitus (5/7, 71.43%), vertigo (5/7, 71.43%), otorrhea (4/7, 57.14%), otalgia (3/7, 42.86%), and headache (2/7, 28.57%). Patient 4 and Patient 5 developed intracranial haemangioblastomas during gestation. The preoperative FN function was classified as H-B I in 1 patient, II in 1 patient, III in 1 patient, IV in 1 patient, and VI in 3 patients. The clinical data of the 7 patients are detailed in Table 2 and Table 3. Table 4 outlines the clinical and pathological differences between two patients with ELST in the context of VHL disease. Patient 1, with a confirmed *VHL* gene mutation, presents with a vascular immunophenotype and benign systemic findings, including a renal cyst that may evolve into ccRCC. Patient 2 demonstrates an epithelial immunoprofile, bilateral vestibular schwannomas, and RCC, indicating higher malignant potential and broader systemic involvement. These distinctions highlight the phenotypic variability of VHL-associated tumors and the importance of integrated surveillance and multidisciplinary care.

### 3.2. Radiological Characteristics

**Figure 2:**
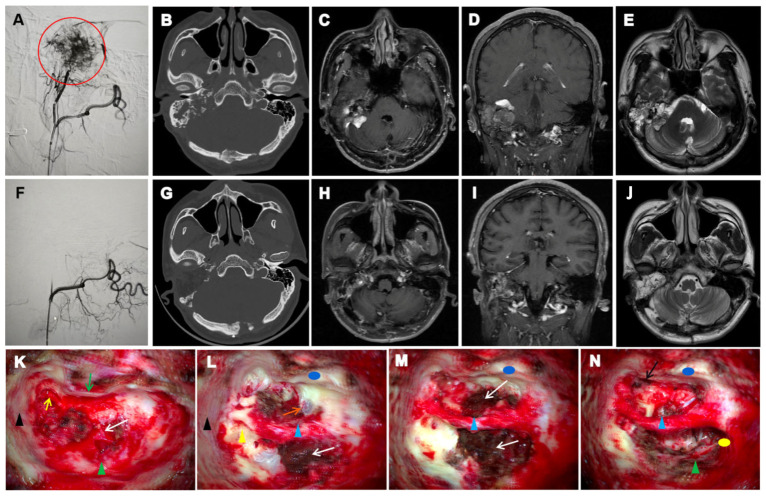
Preoperative and postoperative imaging and intraoperative findings of ELST in case 1. (**A**): DSA shows that the lesion is primarily supplied by branches of the right external carotid artery. A dense vascular nidus is visualized with a clustered appearance (red circle). (**F**): Post-embolization DSA demonstrates a marked reduction in lesion vascularity. Residual perfusion is observed in several small feeding branches, with mild residual enhancement at the lesion margin. (**B**): Axial CT demonstrates erosive bone destruction involving the right petrous bone and mastoid, with expansion of the IAC. Peripheral ring-like calcification and spiculated calcifications within the tumor matrix are evident. (**G**): Postoperative CT shows changes consistent with complete tumor resection. The surgical cavity has well-defined margins. (**C**–**E**): Preoperative contrast-enhanced T1-weighted MRI reveals a heterogeneously enhancing, irregular lesion involving the right petrous bone, ELS, mastoid, and IAC, partially extending into the CPA. T2-weighted images show a hyperintense lesion with irregular peripheral enhancement. (**H**–**J**): Postoperative contrast-enhanced T1-weighted MRI shows postoperative changes in the right petrous bone. Patchy enhancement is seen within the surgical cavity, likely representing postoperative scar tissue. No definitive signs of recurrence are observed. (**K**–**N**): Intraoperative views during ELST resection. Tegmen tympani (black triangle); Short process of the incus (yellow arrow); Posterior wall of the external auditory canal (green arrow); Anterior wall of the external auditory canal (blue circle); Dura of the posterior cranial fossa (green triangle); Internal carotid artery (orange arrow); Pharyngeal orifice of the Eustachian tube (black arrow); Residual bony labyrinth (yellow triangle); FN (blue triangle); ELST (white arrow); Posterior fossa dura (green triangle); Surface of the jugular bulb (yellow circle).

All seven patients in our cohort exhibited characteristic radiological features of VHL-associated ELSTs. Ipsilateral temporal bone osteolytic destruction was observed in all cases (7/7, 100%), with a “moth-eaten” or honeycomb-like pattern present in six patients (6/7, 85.7%). Crest-like or granular calcifications were identified in five cases (5/7, 71.4%), and residual bone formation was noted in four cases (4/7, 57.1%). CT localization revealed that the tumour centre was situated in the vestibular aqueduct operculum region in six patients (6/7, 85.7%). Early-stage lesions (3/7, 42.9%) showed periaqueductal osteolysis with preserved surrounding architecture, facilitating origin identification. In contrast, advanced cases (4/7, 57.1%) demonstrated extension into the jugular foramen or subdural space, which obscured the primary site. MRI findings complemented CT observations. A ring-like calcified rim was visible on CT in five patients (5/7, 71.4%), while ridge-like calcifications within the tumour stroma were seen in four (4/7, 57.1%). Hyperintense peripheral tumour margins and flow voids on T1-weighted imaging were present in six cases (6/7, 85.7%), and heterogeneous signal intensity on T2-weighted imaging was observed in all patients (7/7, 100%). Representative radiological features of Patient 1 are shown in Figure 2.

### 3.3. Surgical Approaches and Outcomes

All 7 patients underwent surgical resection by ELST, and 5 of them underwent digital subtraction angiography (DSA) and arterial embolization before surgery. Patient 1 underwent proactive staged surgical treatment. The first stage involved ELS resection in our department (Figure 2), followed by neurosurgical intervention in the second stage. Due to the highly vascular and hemorrhagic nature of the lesion, intraoperative imaging was compromised by blood interference, resulting in suboptimal clarity. Tumour resection in Patients 2–7 was performed using different surgical approaches. Partial residual tumour was observed in Patient 7 because of intraoperative haemorrhage. Patients 2 and 4 received cochlear implants to improve hearing, which were later removed for MRI because of tumour recurrence. Postoperatively, the FN function remained unchanged in all patients with preoperative H-B I–IV status. Among those with H-B VI status, two underwent great auricular nerve grafting, resulting in recovery to H-B IV classification, whereas one received hypoglossal–FN anastomosis and recovered to H-B V classification.

### 3.4. Genotype-Phenotype Features

The literature and our cases included 12 patients (Table 5). The characteristics were as follows. Exon 1 mutations (5 patients): predominantly female, often right-sided tumours; common tumour types, such as cranial and spinal haemangioblastomas; some with retinal haemangioblastomas. Exon 2 mutation (1 male patient): cranial haemangioblastoma. Exon 3 mutations (6 patients): tumour types included retinal, spinal, and cranial haemangioblastomas, and various concomitant systemic diseases were present. Eight patients had a family history, mainly those with Exon 1 or 3 mutations. Exon 3 mutation inheritance demonstrated clear parent-offspring transmission, particularly in patients with RCC and pheochromocytomas.

## 4. Discussion

### 4.1. Demographic and Clinicopathological Characteristics

Our study comprehensively details the demographic and clinicopathological features of VHL disease-associated ELSTs, confirming established patterns. The mean otologic symptom onset age (22.43 ± 8.68 years) aligns with studies showing that VHL disease-associated ELSTs occur earlier than those in sporadic cases (mean of 29 vs. 52 years) [2,21,22]. The prevalence of ELSTs in our VHL registry (7/75 cases, 9.33%) falls within the wide range of previously reported rates (3.6% [23] to 11–16% [24]). This variability may be attributed to several factors: our institution’s tertiary referral status could introduce selection bias through complex case concentration; the diagnostic challenge posed by the tumor’s indolent progression of the tumour, and the confounding effect of undiagnosed VHL disease in presumed sporadic cases (39% of sporadic ELSTs harbor germline VHL mutations [23]). Notably, only 32% of VHL disease-associated ELSTs manifest as initial disease presentations [23], with many cases remaining clinically silent until advanced stages, further complicating epidemiological comparisons.

### 4.2. Clinical Presentation and Diagnostic Challenges

The distinct clinical profile of our cohort with VHL disease-associated ELSTs is characterized by the prevalence of SNHL in 100% of cases, FN paralysis (6/7, 85.71%), tinnitus (5/7, 71.43%), and vertigo (5/7, 71.43%). Meta-analytic data on sporadic ELSTs report rates of severe or profound SNHL in 88.7% of patients, vertigo in 42.0%, and tinnitus in 61.8%, and a notably lower incidence of FN paralysis at only 30.6% [25]. This disparity, particularly in terms of FN involvement (85.71% vs. 30.6%), likely reflects both the more aggressive nature of VHL disease-associated tumors and consequences of diagnostic delays (median symptom duration 3.5 years) in our cohort.

The diagnostic challenge is compounded by the ability of the tumour to cause significant functional impairment (particularly SNHL, with mean onset at 22 years, which is acute in 43% of cases [26]) despite its histologically indolent growth pattern. Other studies have shown that vestibular symptoms may precede radiological detection [27], although the clinical utility of advanced vestibular testing remains unproven. The frequent misdiagnosis of ELST-related otologic symptoms such as Meniere’s disease underscores the importance of considering ELST in differential diagnoses, particularly in young patients with apparent “Meniere” symptoms and VHL risk factors.

### 4.3. Pathological Manifestation

Histopathology confirmed a characteristic papillary-glandular architecture with a cuboidal epithelial lining, haemorrhage, and necrosis [2]. Immunohistochemically, consistent CK positivity (6/6 cases) confirmed epithelial differentiation. Variable CD56 (3/6) and vimentin (2/6) expression indicated retained neuroectodermal features and biphasic potential, respectively. The uniformly low Ki-67 index (<5%) is consistent with the indolent behaviour of the tumour. Importantly, the observed immunophenotypic heterogeneity—namely, variable GFAP (Patients 2, 4, and 6), CD31/CD34 (Patients 1 and 2), and S-100 (Patients 5 and 7) expression—suggests the existence of distinct clinical genomic ELST subtypes, warranting further investigation.

### 4.4. Therapeutic Considerations and Outcomes

Surgical resection remains the mainstay treatment [28]. DSA was recommended for all patients prior to tumor resection to assess vascular supply and guide embolization. The blood supply mainly arises from the branches of the external carotid artery, including posterior auricular artery, occipital artery, ascending pharyngeal artery, middle meningeal artery or facial artery. Several transcranial tumors can also be supplied from the branches of the internal carotid artery or vertebral artery, such as the posterior inferior cerebellar artery. The vascular supply arises from one or more arteries. Flexible microcatheters (e.g., Echelon, Marathon) with floppy tips and hydrophilic coatings are employed to precisely access the distal segments of feeding arteries, enabling superselective catheterization and minimizing the risk of non-target embolization. These devices, in combination with controllable guidewires (e.g., Synchro), facilitate safe navigation through the complex vascular anatomy of the petrous bone region and help avoid inadvertent entry into dangerous anastomoses, such as those involving the meningeal or ophthalmic arteries. Potential risks of embolization include cranial nerve injury, vessel spasm, vascular perforation, and incomplete devascularization. These complications can be effectively mitigated through real-time fluoroscopic guidance, test injections to verify flow dynamics, and careful avoidance of embolization near critical neurovascular structures. Furthermore, gentle manipulation of microcatheters, heightened awareness of vessel tortuosity, and avoidance of high-pressure injections are essential technical precautions to reduce the likelihood of vessel spasm or perforation. Among the seven cases, five underwent DSA, including all type-II ELSTs, all using Polyvinyl-alcohol (PVA) particles. Two patients (Patients 4 and 5) declined DSA due to financial constraints, although it was clinically advised. Preoperative DSA proved essential in identifying marked hypervascularity and planning embolization. Despite embolization, four patients (Patients 2, 3, 6, and 7) still required intraoperative blood transfusion, highlighting the technical challenges associated with managing highly vascular tumors.

Although several staging systems have been proposed to guide surgical approaches and prognostic evaluation, reported rates of tumor recurrence or residual disease range from 3% to 14%. However, in our clinical experience, the staging systems proposed by Bambakidis [29] and Schipper [30], while informative, show limitations in practical application. Based on our 2022 publication involving long-term follow-up of 22 ELST cases from a single center [9], we found no significant differences between Schipper’s type A and type B tumors in terms of clinical presentation, recurrence/residual rates, or surgical requirements. Subsequent studies have indicated that SNHL associated with ELST is primarily linked to hemorrhage and endolymphatic hydrops within the sac [21], rather than the extent of tumor spread. Accordingly, we simplified and restructured the staging system type I and type II. This revised classification demonstrated strong prognostic value—none of the type I cases showed recurrence or residual disease, whereas type II tumors had a recurrence/residual rate of 31.3% [9]. These findings are consistent with literature reports that gross total resection (GTR) rates are influenced by tumor size and anatomical location [24]. Early intervention is therefore critical for preserving hearing and improving the feasibility of GTR [5,24]. We recommend a transmastoid approach for type I tumors due to their limited invasion. In contrast, type II tumors require a more extensive surgical corridor, with subtotal petrosectomy as the baseline approach. If the tumor involves the middle ear cleft or jugular foramen, cavity obliteration or a Fisch A infratemporal fossa approach with proximal sigmoid sinus ligation should be considered, respectively. To minimize intraoperative bleeding, tumor entry should be avoided until extracapsular dissection has interrupted the blood supply. Rapid removal of the tumor and invaded tissue is also advised to shorten bleeding duration. In summary, our proposed type I/II classification system offers greater clinical utility in both prognostic assessment and surgical planning and contributes to more effective intraoperative bleeding control strategies.

Reconstruction of FN function represents one of the central challenges in surgical management. In our cohort, functional outcomes ranged from H-B I to V. The primary surgical objective was to achieve GTR while striving to preserve or reconstruct FN function. Among the three patients who presented with preoperative H-B VI, all demonstrated varying degrees of postoperative improvement following nerve reconstruction techniques. Although the preservation of hearing—defined as maintaining measurable acoustic hearing thresholds postoperatively—is generally unachievable beyond early-stage cases, CI may offer a viable auditory rehabilitation strategy in bilateral ELST patients. This recommendation is based on practical experience from two patients in our series. Given the high risk of systemic tumour recurrence associated with this disease entity, lifelong MRI surveillance is essential. Therefore, the use of MRI-compatible CI devices is critical to ensure ongoing monitoring without compromising imaging quality.

For inoperable or residual ELSTs, stereotactic radiosurgery serves as a salvage option, although its efficacy is controversial [2,31,32]. While case reports note postradiation reduction in tumour volume and decreased enhancement, long-term studies have shown that radiation provides limited durable control, with progression rates similar to the natural history of untreated lesions [33]. Prophylactic radiotherapy is reserved for tumours unsuitable/unsafe for resection [34]. In our study, Patient 4 (irradiated elsewhere) had recurrence within one year (reaching 10 cm); concurrent pregnancy complicated the effect assessment because of potential hormonal influence. Notably, ELSTs have a low recurrence rate after resection, but multisystemic VHL disease manifestations often pose life-threatening risks. This necessitates comprehensive, lifelong surveillance even after successful ELST management. Recently, targeted therapies (e.g., anti-VEGF antibodies and HIF-1α inhibitors [35]) have offered new hope; however, no ELST-specific agents have been approved, and pharmacological options remain limited.

Currently, there is no literature supporting the routine use of robotic surgery for the resection of ELST. These tumors are typically located deep within the petrous bone, adjacent to critical structures such as cranial nerves, the brainstem, and major vessels. Their surgical management requires high-precision microsurgical techniques and multi-angled drilling, which exceed the current reach and stability capabilities of robotic systems in complex skull base procedures.

### 4.5. Genetic and Pregnancy-Related Considerations

The profound genetic heterogeneity of VHL disease fundamentally dictates clinical management strategies for associated ELSTs. As evidenced by the established literature, VHL mutations are distinctly distributed: missense (44%), frameshift (6%), nonsense (12%), in-frame indels (35%), and splice site variants (3%) [7]. Our consolidated cohort of 12 patients (Table 5), which included both the literature review and original cases, revealed critical exon-specific phenotypic patterns. Exon 1 mutations (n = 5) predominantly affected females (100%). Exon 3 mutations (n = 6) were associated with systemic comorbidities (83.3%). Patients with Exon 3 mutations showing explicit parent-offspring transmission of RCC/pheochromocytoma-associated mutations. Molecular analysis of three index cases revealed exclusive missense mutations aligning with VHL type 2 phenotypes. While Exon 3 α-domain mutations (c.485G>T/Patient 1; c.499C>T/Patient 6) directly compromise HIF-α binding, the Exon 1 β-domain mutation c.194C>G (Patient 7) manifested unusually aggressive, early-onset ELSTs with haemangioblastomas—diverging from typical mild Exon 1 phenotypes. This suggests that Exon 1 β-domain missense mutation c.194C>G may uniquely destabilize pVHL, expanding the pathogenic scope of Exon 1 missense variants. Collectively, these genotype-phenotype features establish a molecular framework for predicting VHL disease-associated ELSTs prognosis [36,37]. Confirmatory genetic diagnosis mandates lifelong multisystem surveillance and enables prenatal prevention of mutation transmission.

**Figure 3:**
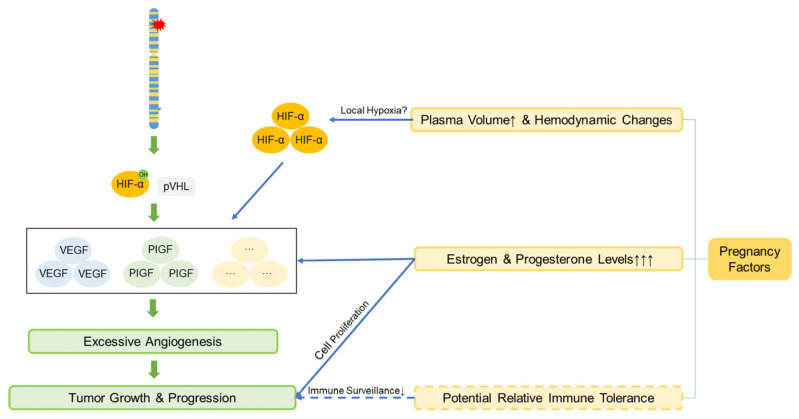
The mechanism by which pregnancy promotes the growth and progression of VHL-associated tumors. Pregnancy-associated factors synergize with the increased HIF caused by *VHL* gene mutations, accelerating angiogenesis and thereby promoting tumor growth and progression. The solid lines represent the established mechanisms, while the dashed lines represent the hypothesized mechanisms. Definitions of abbreviations are provided in the main text.

In patients with VHL disease, median survival exhibits notable sex- and region-related differences: internationally, the median survival is 67 years for males and 60 years for females [38], whereas in China, males have a median survival of 62 years and females 69 years [39]. Some studies show that female patients have shorter life expectancies (48.4 vs. 59.4 years) [2,28]; however, the underlying mechanisms remain unclear and require further study. Pregnancy management in VHL patients is complex. Although prospective studies have indicated that pregnancy itself is not an independent risk factor for new manifestations and should not restrict fertility and that haemangioblastoma symptoms often coincide with childbearing years and may lead to misattribution [40,41], our study revealed symptoms developing postgestation in 2 pregnant patients. Notably, Patient 4 was newly diagnosed with intracranial haemangioblastoma during pregnancy, which is consistent with the findings of Frantzen et al. [42]. These findings suggest potential associations with pregnancy-related physiological changes beyond genotype: (1) increased blood volume and elevated tumour venous pressure may promote expansion or oedema. (2) Pre-existing VHL-related HIF dysregulation, causing HIF-α accumulation and VEGF upregulation, may be exacerbated by placental HIF signalling involved in normal vascular remodelling [43], potentially aided by PlGF/VEGFR-1 signalling [44]. (3) Potential relative immune tolerance enables tumour cells to evade immune surveillance. While VHL-related HIF abnormalities raise theoretical preeclampsia concerns, enhanced prenatal surveillance is unsupported [45]. A possible pathway diagram is shown in Figure 3.

CNS hemangioblastomas are the leading cause of death, particularly when located in critical regions such as the brainstem or cerebellum, where complications like hemorrhage or elevated intracranial pressure can be fatal. RCC ranks second among fatal manifestations, especially when diagnosis is delayed or metastasis occurs, leading to poor prognosis. Other lesions, including pancreatic tumors or cysts, pheochromocytomas, ELSTs, and reproductive system abnormalities—are relatively common in VHL patients but contribute less directly to mortality. Pancreatic lesions are predominantly benign serous cystadenomas with low malignant potential, although a minority of pancreatic neuroendocrine tumors may exhibit aggressive behavior (8–17%) [20]. Pheochromocytomas are typically benign but can secrete excessive catecholamines, triggering hypertensive crises and potentially fatal cardiovascular or cerebrovascular events if not promptly managed. ELST primarily affects auditory and vestibular function and are rarely life-threatening. Reproductive system lesions, such as papillary cystadenomas of the epididymis or broad ligament, are generally benign and exert minimal impact on survival. While these manifestations are not primary causes of death, their potential complications and impact on quality of life. Multidisciplinary team (MDT) management is essential for optimizing VHL disease-associated ELSTs care and integrating expertise across specialties for comprehensive diagnosis, treatment, pregnancy management, and rehabilitation. For instance, preoperative pheochromocytoma screening is crucial for preventing perioperative hypertensive crises. Additionally, because of the risk of recurrence, the use of MRI-compatible CI devices is imperative. Future research priorities include (1) developing risk stratification models combining anatomic and molecular profiles; (2) prospectively evaluating belzutifan and other targeted therapies with surgery/radiotherapy; (3) providing long-term data to optimize surveillance; and (4) investigating vestibular monitoring for early ELST detection. Large-scale and mechanistic studies are needed to overcome survival barriers and improve functional and lifespan outcomes.

On the basis of the literature on VHL-associated ELSTs, our study provides several novel insights and recommendations. First, we observed a markedly high incidence of FN paralysis (85.71%) in our cohort, significantly exceeding the rate of 30.6% reported in meta-analyses of sporadic ELST, indicating a more aggressive otologic phenotype. Second, we propose that pregnancy may stimulate tumour growth and hypothesize that pregnancy-related physiological changes could modulate VHL tumour behaviour. This perspective diverges from those of previous reports and further investigation warrants. Third, we are the first to recommend that hearing rehabilitation in these patients should account for the genetic nature of the disease and the high risk of developing new CNS haemangioblastomas and renal tumours. Long-term MRI surveillance is essential; therefore, MRI-compatible CI devices should be considered. Finally, we conducted the first systematic review of previously reported cases with both genotypic and phenotypic data, these genotype–phenotype features have not been previously quantified. These findings and recommendations are highly relevant for optimizing surveillance and management strategies in patients with VHL disease -associated ELSTs.

## 5. Conclusions

VHL disease-associated ELSTs often present with otologic manifestations such as SNHL and FN paralysis, in addition to distinct phenotypic profiles linked to specific exonic mutations. Surgical excision remains the cornerstone of curative treatment. A thorough genetic evaluation facilitates risk stratification, long-term surveillance, and informed prenatal decision-making. Optimizing clinical outcomes requires a multidisciplinary approach.

## Figures and Tables

**Table 1:** Indications and Findings of Complementary Imaging Modalities in VHL disease-associated ELSTs.

Imaging Modality	Primary Indications	Typical Findings	Clinical Utility
HRCT	Assess bony erosion, tumor boundaries, and preoperative planning	ipsilateral temporal bone “moth-eaten” or honeycomb-like osteolytic destruction with crest-like/granular calcifications and residual bone formation	CT localization typically revealed tumour centres in the vestibular aqueduct operculum region, with early-stage lesions showing periaqueductal osteolysis and preserved surrounding architecture that facilitated origin identification. In advanced cases, the jugular foramen or subdural extension often obscured primary site determination.
MRI	Early screening, Lesion localization and classification, tumor relationship with the inner ear, brainstem, and cranial nerves, postoperative follow-up and long-term surveillance	hyperintense peripheral tumour margins and flow voids on T1-weighted imaging; and heterogeneous signal intensity on T2-weighted imaging.	Superior soft tissue contrast allows early detection of subclinical lesions, precise delineation of tumor extent, and assessment of involvement of critical neurovascular structures. Essential for surgical planning and long-term surveillance, especially in bilateral or recurrent VHL-associated cases.
DSA	Assess vascular supply and guide preoperative embolization	Tumor blush from external carotid artery branches; occasionally supplied by internal carotid or vertebral artery branches	Defines feeding arteries; essential for embolization planning and minimizing intraoperative bleeding risk

**Table 2:** Preoperative Clinical Data of 7 VHL-associated ELST Patients.

Patient	Age of Onset (Year)	Sex	Otologic Symptoms and H-B Grade (Pre/Post)	Family History and Mutation	Abnormal Findings
1	27	M	Otorrhea, FN paralysis, Tinnitus, Complete deafness; H-B III/H-B III (R)	N; c.485G>T	Brain haemangioblastoma, multiple hepatic haemangiomas; Pancreatic cysts; Parotid nodules; Thyroid nodules (Bethesda II), Right vocal cord fixation; Left retinal haemangioblastoma, Left Renal cyst
2	27	M	Vertigo, Tinnitus, Otalgia, FN paralysis (R), Complete deafness (B); H-B VI/H-B V (R)	P; Not tested	Bilateral vestibular schwannomas, cranial hemangioblastomas, Pancreatic cystadenoma; Renal mass; Hyperechoic nodule in the left hepatic lobe
3	29	F	Vertigo, Tinnitus, Otalgia, FN paralysis, Complete deafness; H-B II/H-B II (L)	P; Not tested	Hepatic haemangiomas, Pancreatic cysts, right RCC (Fuhrman I-II), Left renal mass, Bilateral renal cysts
4	10	F	vertigo, Tinnitus, Complete deafness; H-B I/H-B I (B)	P; Not tested	Bilateral renal cysts, Pancreatic tail cyst; right retinal haemangioblastoma, brain haemangioblastoma
5	33	F	Headache, Vertigo, FN paralysis, Complete deafness; H-B IV/H-B IV (L)	N; Not tested	Cerebellar haemangioblastoma
6	17	F	Tinnitus, Otorrhea, FN paralysis, Complete deafness H-B VI/H-B IV (L)	P; c.499C>T	Right retinal haemangioblastoma
7	14	F	Otalgia, Otorrhea, Vertigo, FN paralysis, Complete deafness; H-B VI/H-B IV (L)	P; c.194C>G	Right retinal haemangioblastoma, T1 intramedullary haemangioblastoma, Cerebellar vermis haemangioblastoma

M: Male; F: Female; L: Left; R: Right; P: Positive; N: Negative.

**Table 3:** Surgical data of 7 VHL-associated ELST Patients.

Patient	Tumor Blood Supply	Immunohistochemistry	Surgical History and Outcome
1	Right external carotid artery occipital branch	CK (+), CD31(+), CD34(+), D2-40(−), Ki-67(+3%)	Neurosurgery (2005, 2013, 2024; Other hospital): Brain haemangioblastoma resection ENT (2024, our centre): Subtotal temporal bone resection, FN rerouting (R) Neurosurgery (2025, our centre): Cerebellar haemangioblastoma resection Follow up (2025.8, our centre): No evidence of ELST recurrence.
2	Right external carotid artery	TTF-1 (−), TG (−), CD34 (+), CK7 (+), CK (+), S-100 (−), P63 (−), Ki-67 (+3%), Syn (−), CgA (−), EMA (+), GFAP (−)	Radiation Oncology (2007, Other hospital): Bilateral vestibular schwannoma treated with Gamma Knife ENT (2013, our centre): CI (R) Neurosurgery (2016, 2017, Other hospital): Resection of cranial hemangioblastomas ENT (2019, our centre): Subtotal temporal bone resection, hypoglossal-FN anastomosis (R), cochlear implant explantation (R) ENT (2021, our centre): CI and cochlear implant explantation (L) ENT (2023, our centre): Subtotal temporal bone resection (R) Follow up (2025.8, our centre): No evidence of ELST recurrence. Underwent Phase III clinical trial for RCC (Other hospital).
3	Left carotid artery posterior auricular branch; Sella turcica area shows round tumor-like staining	Microscopy: Partial papillary structures, cystic wall lined by monolayer cuboidal epithelium	Became pregnant in 2001, 2007 ENT (2013, our centre): Subtotal temporal bone resection (L) Urology (2014, our centre): Right nephrectomy Follow up (2025.8, our centre): No evidence of ELST recurrence.
4	-	GFAP (+), CK (+)	ENT (2013, our centre): Extended retrolabyrinthine approach + CI (R) Neurosurgery (2014, Other hospital): hemangioblastoma resection + radiotherapy Got pregnant in 2016 (hemangioblastoma enlargement to 10 cm within 1 year) ENT (2016, our centre): cochlear implant explantation (R) Neurosurgery (2016, Other hospital): Craniospinal hemangioblastoma resection Follow up (2025.8, our centre): No evidence of ELST recurrence.
5	-	TTF-1 (−), S-100 (scattered cells +), Syn (−), CK5 (+), Ki-67 (+1%), TG (−), PAX-8 (partial), CD56 (+), Vimentin (+)	Became pregnant in 2005, 2016 Neurosurgery (2016&2017, Other hospital): Cerebral haemangioblastoma resection ENT (2019, our centre): Subtotal temporal bone resection (L) Neurosurgery (2024, our centre): Midline approach cerebellar haemangioblastoma resection Follow up (2025.8, our centre): No evidence of ELST recurrence.
6	Left external carotid artery; Intracranial presence of 3 aneurysms	TTF-1 (−), Syn (−), CK (+), Ki-67 (+<5%), TG (−), CD56 (+), Vimentin (+), GFAP (−), PAS (+)	ENT (2010, Other hospital): Left mastoidectomy ENT (2011, our centre): Extended retrolabyrinthine approach, great auricular nerve grafting (L) Follow up (2012.10, our centre): MRI revealed residual cerebellopontine angle tumour Follow up (2025.8, our centre): Lost to follow-up
7	Left external carotid artery posterior auricular branch	TTF-1 (−), S-100 (+), Syn (−), CK (+), Ki-67 (−), CD56 (+), Vimentin (−), EMA (−), CgA (−)	Neurosurgery (2011, our centre): T1 intramedullary haemangioblastoma resection ENT (2011, our centre): Subtotal temporal bone resection, great auricular nerve grafting (L) Neurosurgery (2013, our centre): Cerebellar vermis haemangioblastoma resection Follow up (2025.8, our centre): Lost to follow-up

L: Left; R: Right.

**Table 4:** Comparative Summary of Case 1 and Case 2.

Dimension	Case 1	Case 2	Clinical Implication
Age/Sex	27/Male	27/Male	Comparable demographic background
Otologic Symptoms and H-B Grade	Otorrhea, facial nerve paralysis, tinnitus, complete deafness (right); H-B III/III	Vertigo, otalgia, tinnitus, facial nerve paralysis (right), complete bilateral deafness; H-B VI/V	Case 2 presents with more severe bilateral auditory and FN involvement
Family History/Genetic Testing	No family history; *VHL* gene mutation confirmed (c.485G>T, exon 3)	Positive family history: genetic testing not performed	Case 1 is genetically confirmed VHL; Case 2 is clinically suspected but genetically unverified
Vestibular Schwannomas	Absent	Bilateral vestibular schwannomas (treated with Gamma Knife)	Case 2 shows broader cranial nerve involvement, possibly indicating *NF2*-like overlap or VHL variant
Renal Findings	Left renal cyst	Renal mass enrolled in Phase III carcinoma trial	Case 1 presents with a benign renal cyst, which may progress to clear cell renal cell carcinoma (ccRCC) over time; Case 2 has confirmed renal malignancy requiring systemic therapy
Pancreatic Lesions	Pancreatic cysts	Pancreatic cystadenoma	Cystadenoma in Case 2 may carry higher neoplastic potential. However, the cystic lesion in Case 1 carries an estimated 8–17% risk of progression to pancreatic neuroendocrine neoplasm (PNEN) [20].
Other Systemic Findings	Parotid and thyroid nodules (Bethesda II), left retinal hemangioblastoma, right vocal cord fixation	Hyperechoic hepatic nodule	Case 1 shows multi-organ benign involvement; Case 2 has fewer benign lesions but higher malignant risk
Histopathology	Papillary and glandular epithelial architecture consistent with ELST	Papillary epithelial architecture consistent with ELST	Both cases are histologically consistent with ELST
Immunohistochemistry	CK(+), CD31(+), CD34(+), D2-40(−), Ki-67(+3%) → vascular phenotype	CK(+), CK7(+), EMA(+), CD34(+), Ki-67(+3%); TTF-1(−), TG(−), Syn(−), CgA(−), S-100(−), P63(−), GFAP(−) → epithelial phenotype	Case 1 shows vascular markers possibly reflecting stromal components; Case 2 demonstrates classic epithelial differentiation, resembling ccRCC
Tumor Growth Risk	Low Ki-67; no ELST recurrence; recurrent CNS hemangioblastomas; renal cyst requires long-term surveillance	Low Ki-67; no ELST recurrence; renal carcinoma under active treatment → high systemic risk	Case 1 may develop renal malignancy over time; Case 2 is undergoing systemic therapy
Therapeutic Strategy	Requires comprehensive systemic surveillance; prompt multidisciplinary intervention upon new findings; previously underwent CNS hemangioblastoma resection and ELST surgery	Requires systemic therapy and coordinated multidisciplinary management, including ELST surgery and renal carcinoma treatment	Case 1 emphasizes preventive monitoring and individualized intervention; Case 2 necessitates active systemic treatment and cross-specialty collaboration

**Table 5:** Genotype-Phenotype of 12 Patients Collected through a Literature Review and Our Reports.

Exon	Sex	Age (Years)	Tumor Side	Family History and Mutation	Symptoms	Hemangioblastoma	Other Diseases	Outcome	Reference
1	F	14	L	P c.194C>G	Otalgia, Otorrhea, Vertigo, FN paralysis, HL	Spinal, Retinal	-	6 months after ELST resection: No evidence of ELST recurrence 14 years after ELST resection: Lost to follow-up	Our research
	F	24	Not mentioned	P c.194C>T	HL, middle ear mass	Cranial	-	26 months after ELST resection: recurrent destructive ELST of the middle ear appeared	Skalova et al. [11]
	F	32	R	P IVS1 +1G>A	HL, vertigo, headache	Cranial	-	No evidence of ELST recurrence was noted, although the exact timing of follow-up was not specified.	Rao et al. [12]
	F	42	R	N c.291-292insGCCGCAGCCC	Headache, dizziness	Cranial	-	No evidence of ELST recurrence was noted, although the exact timing of follow-up was not specified.	Yang et al. [13]
	F	34	R	N Uncertain	otalgia, hypoacusia	Cranial	Pancreatic Cyst	14 months after ELST resection: No evidence of tumor recurrence	Minteguiaga et al. [14]
2	M	16	L	N c.394delC	HL, visual impairment, diplopia, headache.”	Cranial, Retinal	Multiple pancreatic cysts	ELST recurrence was noted once, followed by reoperation; no further ELST recurrence has been observed, although the timing of follow-up remains unspecified	Lodi et al. [15]
3	M	27	R	N c.485G>T	Otorrhea, FN paralysis, Tinnitus, HL	Cranial, Retinal	Hepatic Haemangioma, Pancreatic Cyst, Parotid Nodule, Left Renal cyst	6 months after ELST resection: No evidence of ELST recurrence	Our research
	F	17	L	P c.499C>T	Tinnitus, Otorrhea, FN paralysis, HL	Spinal	Gallbladder Polyp	1 year after ELST resection: Showed no progression of residual tumour 14 years after ELST resection: Lost to follow-up	Our research
	F	32	L	P c.639-2C>A	Headache, dizziness, HL	Spinal	Pancreatic Cyst, Pheochromocytoma	2 years after ELST resection: No evidence of ELST recurrence	Jensen et al. [16]
	F	30	R	P Missense Mutation (specific unknown)	vertigo	Cranial, retinal	renal cystic tumours	6 months after ELST resection: No evidence of ELST recurrence	Codreanu et al. [17]
	M	16	L	P (His Father carries same gene mutation, had RCC and bilateral pheochromocytomas) c.712C>T	Tinnitus HL disequilibrium	-	Pheochromocytoma	No evidence of ELST recurrence was noted, although the exact timing of follow-up was not specified	Priesemann et al. [18]
	M	21	L	P c.930delG + D3S1259 LOH	truncal sensory disturbance, left FN paralysis and hypalgia at the level of T6-12, hearing loss	Cranial, spinal and retinal	Bilateral Renal Cysts, Left Clear Cell Renal Carcinoma	No evidence of ELST recurrence was noted, although the exact timing of follow-up was not specified	Kawahara et al. [19]

Age (age at onset of otologic symptoms); M: Male; F: Female; L: Left; R: Right; P: Positive; N: Negative; HL: Hearing loss.

## Data Availability

Data is contained within the article.

## Abbreviations

The following abbreviations are used in this manuscript:

VHL	Von Hippel–Lindau
ELST	Endolymphatic sac tumor
ELS	Endolymphatic sac
MDT	Multidisciplinary team
FN	Facial nerve
HIFs	Hypoxia-inducible factors
RCC	Renal cell carcinoma
PTA	Pure-tone audiometry
HRCT	High-resolution computed tomography
MRI	Magnetic resonance imaging
DSA	Digital subtraction angiography
SNHL	Sensorineural hearing loss
IAC	Internal acoustic canal
CPA	Cerebellopontine angle
H&E	Haematoxylin and eosin
PVA	Polyvinyl-alcohol
PNEN	Pancreatic neuroendocrine neoplasm
CI	cochlear implantation
CNS	Central Nervous System
